# Comparison of subxiphoid and lateral intercostal approaches for video-assisted thoracoscopic extended thymectomy: a retrospective cohort study

**DOI:** 10.3389/fsurg.2026.1802382

**Published:** 2026-06-22

**Authors:** Shuai Shi, Zhenlin Jiang, Zuxin Dong, Junting Chen, Jianhua Yan

**Affiliations:** 1 Department of Cardiothoracic Surgery, The People's Hospital of Dazu, Chongqing, China; 2 Department of Thoracic Surgery, Hunan Provincial People's Hospital, The First Affiliated Hospital of Hunan Normal University, Changsha, Hunan Province, China

**Keywords:** intercostal space, myasthenia gravis, postoperative pain, subxiphoid approach, surgical outcomes, thymectomy, thymoma, video-assisted thoracoscopic surgery

## Abstract

**Background:**

Video-assisted thoracoscopic extended thymectomy (VATET) is widely used for thymoma. This study compared perioperative, neurological, and short-term oncologic outcomes between subxiphoid and lateral intercostal approaches.

**Methods:**

This retrospective cohort study included 168 patients who underwent VATET between January 2020 and May 2024. Patients were divided into the subxiphoid approach group (SA, *n* = 85) and the lateral intercostal approach group (ICA, *n* = 83). Outcomes included intraoperative blood loss, postoperative hospital stay, drainage duration, hospitalization costs, postoperative pain assessed by the visual analogue scale (VAS), complications graded using the Clavien–Dindo classification, histopathological findings, neurological outcomes assessed using Myasthenia Gravis Foundation of America Post-Intervention Status (MGFA-PIS), and thymoma recurrence. Multivariable logistic regression identified factors associated with prolonged hospitalization.

**Results:**

Baseline characteristics were comparable (*P* > 0.05), and histopathological features were similar between groups. Compared with the SA group, the ICA group had lower intraoperative blood loss, shorter postoperative stay, shorter drainage duration, and lower hospitalization costs (all *P* < 0.05). VAS scores on postoperative days 1, 3, and 7 were lower in the SA group (all *P* < 0.001). Complication rates were comparable (8.24% vs. 6.02%, *P* > 0.05). Neurological outcomes in myasthenia gravis were similar between groups. Thymoma recurrence occurred in two patients in each group.

**Conclusion:**

Both approaches are safe and feasible for VATET. The lateral intercostal approach improves operative efficiency, whereas the subxiphoid approach reduces early postoperative pain without affecting neurological or short-term oncologic outcomes.

## Introduction

Thymoma is the most common primary tumor of the anterior mediastinum and originates from thymic epithelial cells. Although thymoma generally exhibits indolent biological behavior, it is clinically significant because of its association with autoimmune diseases—most notably myasthenia gravis (MG)—and its potential for local invasion and recurrence. Surgical resection remains the cornerstone of treatment and is considered the most effective approach for achieving long-term disease control and neurological symptom improvement in patients with or without MG (1, 2). Extended thymectomy, defined as complete removal of the thymus together with surrounding mediastinal adipose tissue between both phrenic nerves, has been widely accepted as the standard surgical principle to optimize oncologic and neurological outcomes (3). Traditionally, median sternotomy was regarded as the gold standard approach for thymectomy, providing excellent exposure of the anterior mediastinum and enabling complete resection of thymic tissue. However, sternotomy is associated with significant surgical trauma, increased postoperative pain, longer hospital stay, delayed recovery, and cosmetic concerns. With the advancement of minimally invasive techniques, video-assisted thoracoscopic surgery (VATS) has increasingly replaced open approaches in the management of thymoma and MG, particularly in early-stage disease (4). Multiple studies have demonstrated that thoracoscopic thymectomy achieves oncologic outcomes comparable to those of open surgery while offering advantages such as reduced intraoperative blood loss, shorter hospitalization, faster postoperative recovery, and improved cosmetic results (5, 6). Consequently, minimally invasive thymectomy has become an important surgical strategy in modern thoracic surgery.

Despite the widespread adoption of thoracoscopic thymectomy, the optimal surgical approach remains controversial. Various thoracoscopic techniques have been developed, including unilateral or bilateral intercostal approaches, subxiphoid and subcostal approaches, as well as robot-assisted procedures. Differences in operative exposure, technical complexity, postoperative pain, and perioperative efficiency have led to ongoing debate regarding the most appropriate approach for extended thymectomy (7). Among these techniques, the lateral intercostal approach and the subxiphoid approach are the two most commonly used and extensively studied strategies in current clinical practice.

The lateral intercostal approach, particularly via the left chest, has been widely adopted due to its familiarity among thoracic surgeons and favorable anatomical exposure. This approach allows clear visualization of the left innominate vein, thymic veins, and pericardiophrenic structures, which are critical for safe dissection and complete thymectomy (8). Several studies have suggested that the superior vena cava lies outside the primary operative field during left-sided procedures, potentially reducing the risk of major vascular injury (9). In addition, the lateral intercostal approach provides a relatively large working space, which may facilitate dissection of the upper thymic poles and cervical adipose tissue, especially in patients with larger tumors or tumors predominantly located in the left mediastinum. These anatomical advantages contribute to the widespread clinical adoption of this technique.

However, the lateral intercostal approach inevitably involves intercostal incisions and manipulation of intercostal nerves, which may contribute to increased postoperative pain and discomfort. Postoperative intercostal neuralgia has been recognized as a clinically relevant issue, particularly in the early postoperative period, and may negatively affect patient recovery and quality of life (10). Furthermore, unilateral approaches often require opening of the contralateral pleura to achieve complete resection, which may increase the risk of pleural-related complications.

In contrast, the subxiphoid approach has emerged as an alternative minimally invasive technique aimed at minimizing intercostal nerve injury. By accessing the anterior mediastinum through an incision below the xiphoid process, the subxiphoid approach provides a midline view of the thymus and bilateral mediastinal structures. This technique allows symmetrical visualization of both phrenic nerves and facilitates *en bloc* resection of the thymus and surrounding adipose tissue (7, 11). Avoidance of intercostal incisions has been associated with reduced postoperative pain and improved early postoperative comfort, making the subxiphoid approach an attractive option from a patient-centered perspective (11). In addition, the midline operative view may facilitate more comprehensive visualization of bilateral mediastinal structures. Several comparative studies have reported that the subxiphoid approach may offer advantages in postoperative pain control and early recovery while maintaining comparable safety and short-term oncologic outcomes when compared with intercostal approaches (10–12). In addition, the supine position used during subxiphoid thymectomy allows rapid conversion to median sternotomy in the event of major intraoperative bleeding, which is considered a potential safety advantage (11). Nevertheless, the subxiphoid approach has certain limitations, including restricted operative space in patients with obesity or a narrow substernal area and a learning curve related to instrument handling and anatomical orientation (7).

Despite increasing interest in the subxiphoid approach, consensus regarding the optimal thoracoscopic strategy for extended thymectomy has not been fully established. Existing studies have reported inconsistent results regarding operative efficiency, postoperative recovery, and perioperative outcomes, likely due to variations in study design, patient selection, and surgeon experience (10–12). Moreover, data comparing postoperative pain trajectories, hospitalization costs, and short-term recurrence between the two approaches in routine clinical practice remain limited.

Therefore, the present retrospective study aimed to compare perioperative outcomes, postoperative pain, complication rates, hospitalization costs, and short-term recurrence between patients undergoing video-assisted thoracoscopic extended thymectomy via the subxiphoid approach and those treated via the lateral intercostal approach. By evaluating both clinical and perioperative indicators, this study seeks to further clarify the relative advantages and limitations of these two commonly employed thoracoscopic techniques and to support individualized surgical decision-making in patients with thymoma.

## Methods

### Ethics statement

This study was conducted in accordance with the Declaration of Helsinki and was approved by the Ethics Committee of The People's Hospital of Dazu, Chongqing (Approval number: 2024-82). Given the retrospective nature of the study and the use of anonymized clinical data, the requirement for written informed consent was waived by the Ethics Committee.

### Study design and patient selection

**Figure 1:**
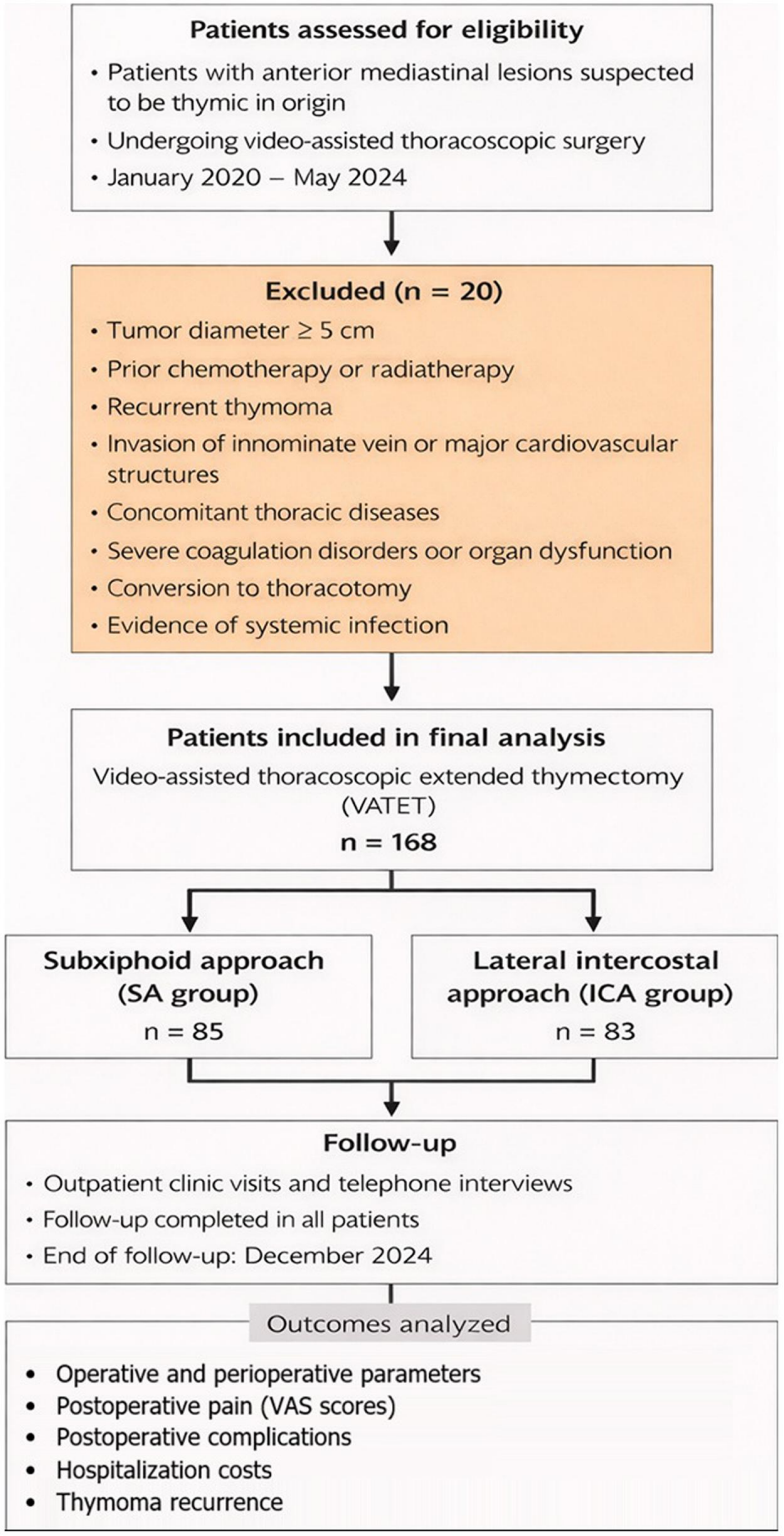
Flow diagram of patient selection and study inclusion. Flow diagram showing patient screening, exclusion, and group allocation. Of 188 patients assessed between January 2020 and May 2024, 168 who underwent video-assisted thoracoscopic extended thymectomy (VATET) were included in the final analysis and allocated to the subxiphoid approach (SA) group or the lateral intercostal approach (ICA) group. All patients completed follow-up.

This study was a single-center retrospective comparative cohort study. Clinical data of consecutive patients who underwent video-assisted thoracoscopic surgery for anterior mediastinal lesions suspected to be of thymic origin at Department of Cardiothoracic Surgery, The People's Hospital of Dazu, Chongqing, between January 2020 and May 2024 were reviewed. A total of 188 patients were initially assessed for eligibility. Patients were eligible for inclusion if preoperative contrast-enhanced chest computed tomography (CT) or mediastinal magnetic resonance imaging (MRI) suggested a thymic-origin lesion, if they had clear surgical indications with stable clinical status, and if the maximum tumor diameter was less than 5 cm. Exclusion criteria included tumor diameter ≥ 5 cm, prior chemotherapy or radiotherapy, recurrent thymoma, invasion of the innominate vein or major cardiovascular structures, concomitant thoracic diseases, severe coagulation disorders or organ dysfunction, intraoperative conversion to thoracotomy, or evidence of systemic infection. Cases requiring intraoperative conversion to thoracotomy were excluded to maintain homogeneity of minimally invasive procedures and to allow accurate comparison between thoracoscopic approaches. No conversions to median sternotomy occurred during the study period. After applying these criteria, 20 patients were excluded, and the remaining 168 patients who underwent video-assisted thoracoscopic extended thymectomy (VATET) were included in the final analysis. According to the surgical approach used, patients were allocated to the subxiphoid approach group (SA group, *n* = 85) or the lateral intercostal approach group (ICA group, *n* = 83) (Figure 1).

### Surgical techniques

**Figure 2:**
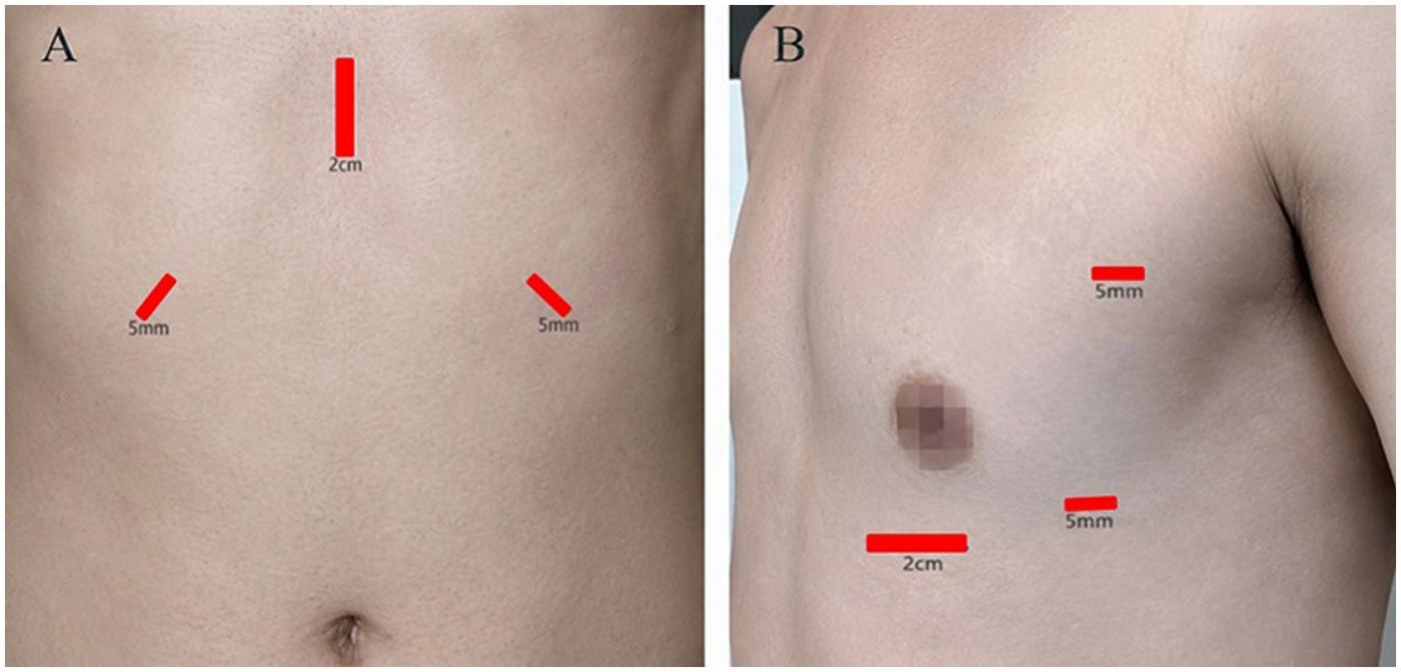
Surgical incision sites for the two thoracoscopic approaches. Representative images showing port placement and incision design for the two thoracoscopic approaches. **(A)** Subxiphoid approach: a 2-cm longitudinal incision inferior to the xiphoid process with two additional 5-mm auxiliary incisions placed bilaterally below the costal margins. **(B)** Lateral intercostal approach: a 2-cm working incision at the fifth intercostal space along the midclavicular line, with two additional 5-mm ports at the third and fifth intercostal spaces along the anterior axillary line.

All procedures were performed by experienced thoracic surgeons proficient in thoracoscopic thymectomy. The same surgical team was involved in both the subxiphoid and lateral intercostal approaches. All participating surgeons had substantial prior experience in minimally invasive thymectomy before the study period. Extended thymectomy was defined as complete removal of the thymus together with surrounding mediastinal adipose tissue located anterior to the great vessels and between the bilateral phrenic nerves. For the subxiphoid approach, patients were placed in the supine position with the back slightly elevated. A 2-cm longitudinal incision was made inferior to the xiphoid process as the observation port, through which a thoracoscope was inserted. Carbon dioxide insufflation was applied at a pressure of 10 cm H₂O to create the operative space. Two additional 5-mm incisions were created bilaterally below the costal margin along the midclavicular line, and an auxiliary 2-cm incision was made below the xiphoid process for operative instruments (Figure 2). After entering the mediastinum, loose retrosternal tissue was dissected to establish the surgical field. Both mediastinal pleura were incised, and dissection proceeded cranially behind the sternum to the inferior border of the left innominate vein. Perithymic adipose tissue was carefully dissected along the anterior borders of both phrenic nerves from caudal to cranial. The thymic upper poles and surrounding adipose tissue were completely mobilized and removed *en bloc*. A drainage tube was placed through the observation port at the conclusion of the procedure.

For the lateral intercostal approach, patients with clearly right-sided tumors underwent a right-sided thoracoscopic approach, whereas all other patients underwent a left-sided approach. In the left-sided approach, patients were placed in the supine position with the left side elevated approximately 30°. A 5-mm incision was made at the fifth intercostal space along the anterior axillary line as the camera port, followed by carbon dioxide insufflation at a pressure of 10 cm H₂O. A 2-cm working port was established at the fifth intercostal space along the midclavicular line, and an additional 5-mm port was created at the third intercostal space along the anterior axillary line. Using an ultrasonic scalpel, adhesions were dissected beginning at the left lower thymic pole. The left costophrenic and pericardial fat pads were removed, and the left phrenic nerve was identified and preserved. The left innominate vein and thymic veins were exposed, with the thymic veins clipped and divided. The contralateral mediastinal pleura was opened, and perithymic adipose tissue was dissected along the anterior border of the right phrenic nerve. The thymic upper and lower poles, along with surrounding mediastinal adipose tissue, were completely resected. A closed thoracic drainage tube was placed through the camera port at the end of the operation.

### Postoperative management and pain assessment

All patients received standardized postoperative analgesia using a patient-controlled intravenous analgesia pump containing sufentanil, which was routinely removed on postoperative day 2. Additional analgesics were administered as needed according to patient symptoms. Postoperative pain was assessed using the visual analogue scale (VAS) on postoperative days 1, 3, and 7, with scores ranging from 0 (no pain) to 10 (severe pain).

### Data collection and outcome measures

Baseline clinical data, including age, sex, body mass index (BMI), myasthenia gravis status, and maximum tumor diameter, were collected. Perioperative outcomes included operation time, intraoperative blood loss, postoperative hospital stay, drainage tube duration, and total hospitalization costs.

Neurological outcomes in patients with myasthenia gravis were evaluated using the Myasthenia Gravis Foundation of America Post-Intervention Status (MGFA-PIS) classification, which categorizes postoperative status into complete stable remission, pharmacologic remission, minimal manifestation, improved and unchanged.

Histopathological evaluation of resected specimens was performed according to the World Health Organization (WHO) classification of thymic epithelial tumors. Tumor stage was determined using the Masaoka–Koga staging system. Resection status was classified as R0 (complete resection with negative margins), R1 (microscopic residual disease), or R2 (macroscopic residual disease).

Drainage tubes were removed when there was no air leak, drainage fluid was serous with a volume of ≤ 100 mL per 24 h, and chest radiography confirmed adequate lung expansion without pleural effusion. Postoperative complications were recorded and graded according to the Clavien–Dindo classification system, which stratifies complications based on the severity of intervention required.

### Follow-up and outcome assessment

Patients were followed through outpatient visits and telephone interviews. Follow-up evaluations included assessment of symptom status, postoperative complications, and imaging studies when indicated. Thymoma recurrence was defined as the appearance of new lesions suggestive of tumor relapse on contrast-enhanced chest CT or MRI during follow-up. In cases of suspected recurrence, additional evaluation with positron emission tomography/computed tomography (PET/CT), biopsy, or reoperation was performed when clinically indicated. Thymoma recurrence during the follow-up period was recorded.

### Statistical analysis

Statistical analyses were performed using SPSS version 24.0. Continuous variables were expressed as mean ± standard deviation and compared between groups using the independent-samples t-test when normally distributed. Categorical variables were presented as frequencies and percentages and analyzed using the chi-square test or Fisher's exact test, as appropriate. Multivariable logistic regression analysis was additionally performed to evaluate factors associated with prolonged postoperative hospital stay. Prolonged postoperative hospital stay was defined as a length of stay greater than the cohort median. Variables entered into the model included surgical approach, age, body mass index, presence of myasthenia gravis, and maximum tumor diameter. A two-sided *P* value of less than 0.05 was considered statistically significant.

## Results

### Patient characteristics

A total of 168 patients who underwent video-assisted thoracoscopic extended thymectomy (VATET) between January 2020 and May 2024 were included in the final analysis. According to the surgical approach, 85 patients were assigned to the subxiphoid approach group (SA group) and 83 patients to the lateral intercostal approach group (ICA group). Baseline demographic and clinical characteristics were well balanced between the two groups. No significant differences were observed in age, sex distribution, body mass index (BMI), prevalence of myasthenia gravis, or maximum thymoma diameter between the SA and ICA groups (all *P* > 0.05), indicating good baseline comparability (Table 1).

**Table 1:** Baseline demographic and clinical characteristics of patients.

Characteristic	SA group (*n* = 85)	ICA group (*n* = 83)	*P* value
Age (years)	52.44 ± 13.45	50.15 ± 13.63	0.274
BMI (kg/m²)	21.58 ± 3.44	20.60 ± 3.07	0.053
Sex, *n* (%)			0.751
Male	44 (51.7)	43 (51.8)	
Female	41 (48.3)	40 (48.2)	
Myasthenia gravis, *n* (%)	46 (54.1)	45 (54.2)	0.344
Maximum tumor diameter (cm)	3.14 ± 1.02	3.12 ± 1.03	0.176

Data are presented as mean ± standard deviation or number (percentage).

SA, subxiphoid approach; ICA, intercostal approach; BMI, body mass index.

### Histopathological characteristics

Histopathological evaluation of the resected specimens revealed comparable tumor characteristics between the two groups. According to the World Health Organization (WHO) classification, the majority of tumors in both groups were type AB, B1, or B2 thymomas. Similarly, the distribution of tumor stages based on the Masaoka–Koga staging system was comparable, with most tumors classified as stage I or stage II. Complete surgical resection (R0) was achieved in the vast majority of patients in both groups, and no statistically significant differences were observed in WHO classification, Masaoka–Koga stage, or resection status between the SA and ICA groups (all *P* > 0.05) (Table 2).

**Table 2:** Histopathological characteristics.

Variable	SA group (*n* = 85)	ICA group (*n* = 83)	*P* value
WHO classification			
Type A	10 (11.8%)	9 (10.8%)	0.91
Type AB	24 (28.2%)	23 (27.7%)	0.94
Type B1	18 (21.2%)	17 (20.5%)	0.89
Type B2	20 (23.5%)	21 (25.3%)	0.82
Type B3	9 (10.6%)	8 (9.6%)	0.88
Other thymic epithelial tumors	4 (4.7%)	5 (6.0%)	0.74
Masaoka-Koga stage			
Stage I	46 (54.1%)	44 (53.0%)	0.90
Stage II	30 (35.3%)	29 (34.9%)	0.95
Stage III	9 (10.6%)	10 (12.0%)	0.79
Stage IV	0	0	—
Resection status			
R0	82 (96.5%)	80 (96.4%)	0.98
R1	3 (3.5%)	3 (3.6%)	0.98
R2	0	0	—

### Neurological outcomes in patients with myasthenia Gravis

Among the 91 patients diagnosed with myasthenia gravis (46 in the SA group and 45 in the ICA group), postoperative neurological outcomes were evaluated using the Myasthenia Gravis Foundation of America Post-Intervention Status (MGFA-PIS) classification. The proportions of patients achieving complete stable remission, pharmacologic remission, minimal manifestation status, or clinical improvement were similar between the two groups, and no statistically significant differences were observed in the distribution of MGFA-PIS categories (all *P* > 0.05) (Table 3). These findings indicate that both surgical approaches provided comparable short-term neurological outcomes in patients with myasthenia gravis.

**Table 3:** Neurological outcomes in patients with myasthenia gravis.

MGFA Post-Intervention Status	SA group (*n* = 46)	ICA group (*n* = 45)	*P* value
Complete stable remission (CSR)	3 (6.5%)	2 (4.4%)	0.82
Pharmacologic remission (PR)	5 (10.9%)	5 (11.1%)	0.74
Minimal manifestation (MM)	12 (26.1%)	11 (24.4%)	0.85
Improved	19 (41.3%)	21 (46.7%)	0.66
Unchanged	7 (15.2%)	6 (13.3%)	0.79

### Comparison of operative and perioperative parameters

**Figure 3:**
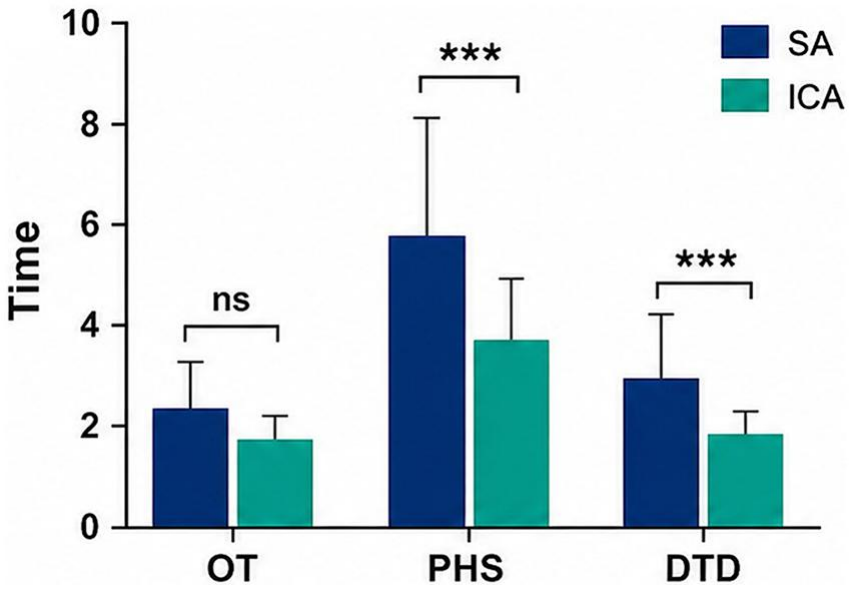
Comparison of operative and perioperative time-related parameters. Comparison of operation time (OT), postoperative hospital stay (PHS), and drainage tube duration (DTD) between the subxiphoid approach (SA) group and the lateral intercostal approach (ICA) group. No significant difference was observed in operation time between groups, whereas postoperative hospital stay and drainage tube duration were significantly shorter in the ICA group. Data are presented as mean ± standard deviation. ns, not significant; ***P < 0.0001.

Perioperative outcomes are summarized in Figure 3. Operation time did not differ significantly between the two groups (*P* > 0.05). However, postoperative hospital stay and drainage tube duration were significantly shorter in the ICA group compared with the SA group (both *P* < 0.001), suggesting a faster postoperative recovery associated with the lateral intercostal approach.

### Intraoperative blood loss and hospitalization costs

**Figure 4:**
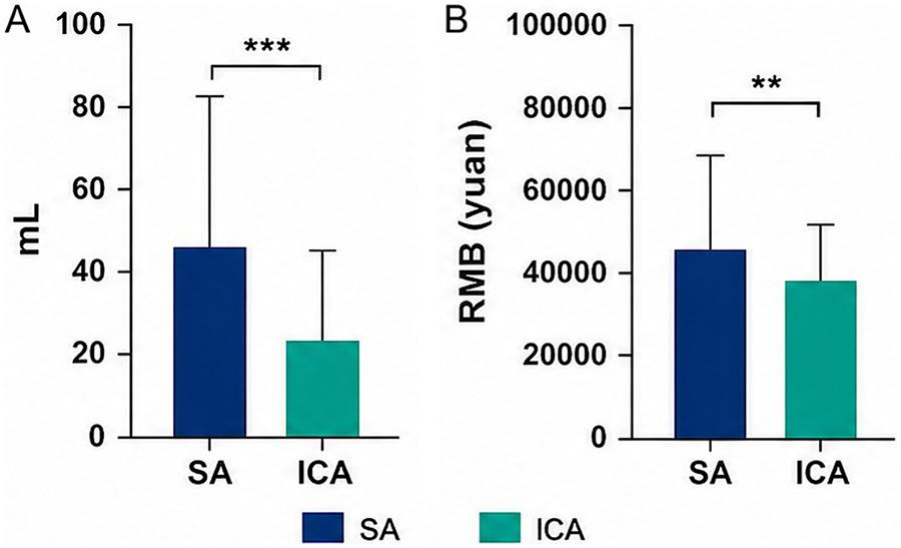
Intraoperative blood loss and hospitalization costs. **(A)** Comparison of intraoperative blood loss between the SA and ICA groups. Blood loss was significantly lower in the ICA group. **(B)** Comparison of total hospitalization costs between the two groups, showing significantly reduced costs in the ICA group. Data are presented as mean ± standard deviation. ***P < 0.0001; **P < 0.001.

As shown in Figure 4A, intraoperative blood loss was significantly lower in the ICA group than in the SA group (*P* < 0.001). Additionally, total hospitalization costs were significantly reduced in patients undergoing the lateral intercostal approach compared with those treated via the subxiphoid approach (*P* < 0.01) (Figure 4B), indicating greater operative efficiency and reduced healthcare resource utilization.

### Postoperative pain assessment

**Figure 5:**
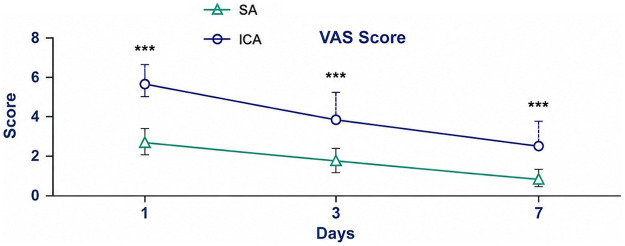
Postoperative pain assessment using visual analogue scale. (VAS) Comparison of postoperative pain scores between the SA and ICA groups assessed using the visual analogue scale (VAS) on postoperative days 1, 3, and 7. VAS scores were significantly lower in the SA group at all time points, indicating superior early postoperative pain control. Data are presented as mean ± standard deviation. ***P < 0.0001.

Postoperative pain was evaluated using the visual analogue scale (VAS) on postoperative days 1, 3, and 7. As illustrated in Figure 5, VAS scores were significantly lower in the SA group than in the ICA group at all assessed time points (all *P* < 0.001), demonstrating superior early postoperative pain control associated with the subxiphoid approach.

### Postoperative complications

Postoperative complications are summarized in Table 4. The overall complication rate was 8.24% in the SA group and 6.02% in the ICA group, with no statistically significant difference between groups (*χ*² = 0.554, *P* = 0.456). Individual complications, including pulmonary infection, pleural effusion, arrhythmia, and poor wound healing, occurred at low and comparable frequencies in both groups.

**Table 4:** Comparison of postoperative complication rates between the two groups.

Complication	SA (*n* = 85), *n* (%)	ICA (*n* = 83), *n* (%)	*P* value
Postoperative pulmonary infection	1 (1.17)	2 (2.41)	0.615
Pleural effusion	2 (2.35)	0 (0.00)	0.497
Arrhythmia	1 (1.17)	2 (2.41)	0.615
Poor wound healing	3 (3.53)	1 (1.20)	0.333
Total complications	7 (8.24)	5 (6.02)	0.456

Values are presented as *n* (%). Percentages are calculated using the group denominators (SA *n* = 85; ICA *n* = 83). *P* values for individual complications were calculated using Fisher's exact test due to small event numbers. The overall comparison of total postoperative complication rates was analyzed using the chi-square test (*χ*² = 0.554).

To further standardize complication reporting, postoperative adverse events were also classified according to the Clavien–Dindo grading system. Most complications were categorized as grade I or grade II events requiring only conservative management or pharmacological treatment, while only a small number required minor interventional procedures (grade IIIa). No grade IIIb, IV, or V complications were observed in either group (Table 5). These findings further confirm the favorable safety profile of thoracoscopic extended thymectomy using either surgical approach.

**Table 5:** Postoperative complications classified by clavien-dindo grade.

Clavien-Dindo grade	SA group (*n* = 85)	ICA group (*n* = 83)	*P* value
Grade I	3 (3.5%)	2 (2.4%)	0.68
Grade II	3 (3.5%)	2 (2.4%)	0.68
Grade IIIa	1 (1.2%)	1 (1.2%)	1.00
Grade IIIb	0	0	—
Grade IV	0	0	—
Grade V	0	0	—
Total	7 (8.2%)	5 (6.0%)	0.46

### Follow-up and thymoma recurrence

All patients completed follow-up through outpatient visits and telephone interviews, with follow-up concluding in December 2024. Thymoma recurrence occurred in 2 patients (2.35%) in the SA group and 2 patients (2.41%) in the ICA group. The overall recurrence rate was low, and no statistically significant difference in recurrence was observed between the two groups (*P* > 0.05). No disease-related mortality occurred during the follow-up period (Table 6). Detailed clinicopathological and surgical characteristics of patients who developed recurrence are summarized in Supplementary Table S1. Owing to the small number of recurrence events (*n* = 4), additional statistical analysis of recurrence-related factors was not performed.

**Table 6:** Follow-up outcomes and thymoma recurrence in the two groups.

Follow-up variable	SA (*n* = 85)	ICA (*n* = 83)	*P* value
Follow-up completion, *n* (%)	85 (100)	83 (100)	—
Follow-up end point	December 2024	December 2024	—
Thymoma recurrence, *n* (%)	2 (2.35)	2 (2.41)	1.00
Patients without recurrence, *n* (%)	83 (97.65)	81 (97.59)	—
Disease-related mortality, *n* (%)	0 (0)	0 (0)	—

SA, subxiphoid approach; ICA, intercostal approach. Follow-up was conducted through outpatient visits and telephone interviews. Recurrence was confirmed by postoperative imaging during follow-up. No statistically significant difference in recurrence rates was observed between the two groups.

### Multivariable analysis of factors associated with prolonged postoperative hospital stay

Multivariable logistic regression analysis was performed to identify independent factors associated with prolonged postoperative hospital stay. After adjustment for age, body mass index, presence of myasthenia gravis, and tumor diameter, the intercostal approach was independently associated with a lower likelihood of prolonged postoperative hospitalization compared with the subxiphoid approach (OR 0.58, 95% CI 0.34–0.97, *P* = 0.038). Tumor diameter was also identified as an independent predictor of prolonged hospital stay (OR 1.29, 95% CI 1.01–1.64, *P* = 0.041), whereas age, BMI, and myasthenia gravis were not significantly associated with this outcome (Table 7).

**Table 7:** Multivariable logistic regression for prolonged postoperative hospital stay.

Variable	OR	95% CI	*P* value
ICA vs SA approach	0.58	0.34–0.97	0.038
Age (per year)	1.02	0.99–1.05	0.14
BMI (per kg/m²)	1.04	0.96–1.13	0.31
Myasthenia gravis	1.41	0.82–2.43	0.21
Tumor diameter (per cm)	1.29	1.01–1.64	0.041

## Discussion

Thymectomy remains the cornerstone of treatment for thymoma and myasthenia gravis, with extended thymectomy widely accepted as the optimal surgical strategy to ensure complete removal of thymic tissue and perithymic adipose tissue (13). With the evolution of minimally invasive surgery, video-assisted thoracoscopic extended thymectomy (VATET) has largely replaced median sternotomy in selected patients, owing to its reduced surgical trauma, faster recovery, and comparable oncologic efficacy (14). However, the choice of thoracoscopic approach remains controversial, particularly between the lateral intercostal and subxiphoid approaches. In this retrospective comparative study, we evaluated perioperative outcomes, postoperative pain, complications, hospitalization costs, and short-term recurrence associated with these two commonly used techniques. The present study demonstrated that both the lateral intercostal and subxiphoid approaches are safe and feasible for VATET, with no significant differences in postoperative complication rates or short-term thymoma recurrence. These findings are consistent with previous reports suggesting that minimally invasive thymectomy, regardless of approach, can achieve satisfactory short-term oncologic outcomes when the principles of extended thymectomy are strictly followed (15, 16). Importantly, no cases of perioperative mortality or phrenic nerve injury were observed in either group, underscoring the safety of both techniques when performed by experienced surgeons. Another important technical consideration relates to exposure of the superior thymic poles and cervical adipose tissue during extended thymectomy. Previous studies have reported that the subxiphoid approach may present challenges in accessing the upper thymic poles, particularly in patients with myasthenia gravis, in whom complete removal of cervical thymic tissue and perithymic fat is considered important for optimal neurological outcomes. This limitation is primarily related to the caudal-to-cranial viewing angle and restricted maneuverability within the superior mediastinum. In our experience, adequate visualization and dissection of the upper poles were achieved through careful traction and meticulous surgical technique; however, this step may be technically demanding and requires sufficient operator experience (7, 17). In addition to perioperative safety, the present study evaluated neurological outcomes in patients with myasthenia gravis using the Myasthenia Gravis Foundation of America Post-Intervention Status (MGFA-PIS) classification. More than half of the patients in our cohort were diagnosed with myasthenia gravis, reflecting the typical clinical indications for thymectomy in patients with thymic tumors. The distribution of MGFA-PIS categories, including complete stable remission, pharmacologic remission, minimal manifestation status, and clinical improvement, was comparable between the two surgical approaches. These findings suggest that both the subxiphoid and lateral intercostal approaches provide similar short-term neurological benefits in patients with myasthenia gravis. Previous clinical studies have similarly reported favorable neurological outcomes following minimally invasive thymectomy when complete thymic and perithymic tissue removal is achieved, supporting the feasibility of thoracoscopic approaches in patients with thymoma-associated myasthenia gravis (5, 12, 16, 18). One of the key findings of this study is that the lateral intercostal approach was associated with greater operative efficiency, as reflected by shorter postoperative hospital stay, reduced drainage tube duration, lower intraoperative blood loss, and decreased total hospitalization costs. These results are in line with previous studies and meta-analyses reporting that the intercostal approach may provide a larger operative field and more flexible instrument maneuverability, thereby facilitating dissection and hemostasis (19, 20). In particular, the left-sided intercostal approach offers favorable anatomical exposure of the left innominate vein and thymic veins, potentially reducing the risk of vascular injury and intraoperative bleeding (21). The observed reduction in hospitalization costs further highlights the potential economic advantages of the lateral intercostal approach, which is increasingly relevant in the context of value-based healthcare. A recent randomized controlled trial by Wang et al. comparing subxiphoid and lateral intercostal thoracoscopic thymectomy for thymoma similarly demonstrated that both approaches are safe and effective, although differences in operative efficiency and postoperative recovery may favor specific approaches depending on clinical circumstances (22). Additionally, a propensity score-matched analysis by Zhao et al. reported comparable oncologic outcomes between the two approaches while highlighting differences in perioperative parameters, findings that are consistent with the results of the present study (23).

In contrast, postoperative pain assessment revealed that patients undergoing the subxiphoid approach experienced significantly lower visual analogue scale (VAS) scores during the early postoperative period. This finding supports the theoretical advantage of the subxiphoid approach in avoiding intercostal nerve injury, which is a well-recognized contributor to postoperative pain following thoracoscopic surgery (24). Several previous studies have similarly reported superior pain control and improved early postoperative comfort with the subxiphoid approach compared with intercostal approaches (25, 26). Experimental and clinical evidence has also demonstrated that avoidance of intercostal nerve compression may significantly reduce postoperative pain, as illustrated by comparative wound pain analyses reported in thoracoscopic surgery studies (27). Reduced postoperative pain may facilitate earlier mobilization, improved respiratory function, and enhanced patient satisfaction, all of which are important components of enhanced recovery after surgery (ERAS) protocols.

Despite these differences in perioperative outcomes and pain control, the two approaches did not differ significantly in terms of postoperative complications. The overall complication rates in both groups were low and comparable, consistent with previously published series of thoracoscopic thymectomy (20, 28). Common complications such as pulmonary infection, pleural effusion, and arrhythmia occurred infrequently and were managed conservatively. Furthermore, when complications were categorized according to the Clavien–Dindo classification, most postoperative events were low-grade complications (grade I–II) requiring only conservative treatment or pharmacological management, while only rare cases required minor interventional procedures (grade IIIa). No severe complications (grade IIIb–V) occurred in either group, further confirming the safety of thoracoscopic extended thymectomy using either surgical approach. Histopathological evaluation also demonstrated comparable tumor characteristics between the two groups. The distribution of WHO thymoma subtypes and Masaoka-Koga tumor stages was similar in both surgical cohorts, and complete resection (R0) was achieved in more than 96% of cases. These findings indicate that both thoracoscopic approaches allow adequate oncologic resection of thymic tumors when appropriate surgical principles are followed. Comparable oncologic adequacy of minimally invasive thymectomy has also been reported in recent clinical series evaluating subxiphoid and intercostal thoracoscopic techniques (29). With regard to oncologic outcomes, thymoma recurrence during the follow-up period was rare and did not differ between the two groups. Although the follow-up duration in this study was limited to the end of 2024, the comparable recurrence rates observed are consistent with the principle that oncologic efficacy depends primarily on the completeness of resection rather than the surgical approach itself (16, 30). Current evidence suggests that minimally invasive approaches, including both subxiphoid and intercostal techniques, can achieve oncologic outcomes comparable to those of open surgery in early-stage thymoma (31). Postoperative adjuvant radiotherapy may also play an important role in reducing the risk of local recurrence in selected patients with thymoma, particularly in cases with advanced Masaoka–Koga stage, aggressive histological subtype, capsular invasion, or incomplete resection margins. Although most patients in the present cohort underwent complete (R0) resection and therefore did not routinely receive postoperative radiotherapy, the potential contribution of adjuvant treatment to long-term local control should be acknowledged when interpreting recurrence outcomes (32). Nevertheless, longer follow-up is required to confirm the equivalence of these approaches in terms of long-term recurrence and survival**.** Detailed clinicopathological characteristics of patients who developed recurrence are summarized in Supplementary Table S1. Multivariable logistic regression analysis further demonstrated that the intercostal approach was independently associated with a reduced risk of prolonged postoperative hospital stay. Tumor diameter was also identified as an independent predictor of postoperative recovery time, suggesting that larger tumors may increase operative complexity and delay postoperative recovery. These findings highlight the importance of individualized surgical planning based on both tumor characteristics and surgical approach.

An alternative strategy advocated by some surgeons is the unilateral left-sided thoracoscopic approach without intentional violation of the contralateral pleura. This technique may preserve contralateral pleural integrity and potentially reduce pleural-related complications while maintaining satisfactory exposure of the thymus and mediastinal fat tissue. However, concerns remain regarding visualization of the contralateral phrenic nerve and completeness of extended thymic and perithymic tissue dissection, particularly in patients with myasthenia gravis. In the present study, the contralateral mediastinal pleura was routinely opened during the lateral intercostal approach to facilitate complete resection. Further comparative studies are warranted to determine whether unilateral approaches can provide equivalent surgical, neurological, and oncological outcomes (11, 12). The choice between the subxiphoid and lateral intercostal approaches should therefore be individualized. The lateral intercostal approach may be preferable in patients where operative efficiency, reduced blood loss, and lower hospitalization costs are prioritized, particularly in cases with lateralized or larger tumors. Conversely, the subxiphoid approach may be advantageous in patients for whom postoperative pain control and early comfort are of primary concern, such as those with myasthenia gravis or compromised respiratory function (25, 33). Surgeon experience and institutional expertise also play a critical role in determining the optimal approach. Although all procedures were performed by experienced thoracic surgeons, differences in individual familiarity and technical proficiency with each thoracoscopic approach may still have influenced perioperative outcomes.

Several limitations of this study should be acknowledged. First, the retrospective single-center design introduces the potential for selection bias, as the choice of surgical approach was influenced by surgeon preference and tumor characteristics. Second, although baseline characteristics were comparable between groups, unmeasured confounding factors may have influenced outcomes. Third, follow-up duration was relatively short for thymoma, which is characterized by indolent biological behavior and the potential for late recurrence. Furthermore, only four recurrence events were observed during follow-up, limiting the statistical strength of recurrence-related comparisons and precluding definitive conclusions regarding long-term oncologic superiority between approaches. Fourth, although neurological outcomes were evaluated using the MGFA-PIS classification, longer follow-up is necessary to assess long-term remission and neurological improvement in patients with myasthenia gravis. Fifth, postoperative pain assessment was limited to the early postoperative period and did not include long-term pain evaluation or quality-of-life analysis. Finally, exclusion of patients requiring conversion to thoracotomy may have introduced selection bias and may limit the generalizability of the findings to more technically complex cases. Although multivariable analysis was performed, residual confounding inherent to retrospective studies cannot be completely excluded. Future prospective multicenter studies with longer follow-up and standardized outcome measures are warranted to further validate these findings (34).

## Conclusion

The present study demonstrates that both the lateral intercostal and subxiphoid approaches are safe and effective techniques for VATET, each with distinct advantages. The lateral intercostal approach offers greater operative efficiency and cost benefits, whereas the subxiphoid approach provides superior early postoperative pain control. These findings support a tailored approach to surgical decision-making, taking into account patient characteristics, tumor features, and surgeon expertise.

## Data Availability

The original contributions presented in the study are included in the article/Supplementary Material, further inquiries can be directed to the corresponding author.
