# Supplementary material for: Comparison of subxiphoid and lateral intercostal approaches for video-assisted thoracoscopic extended thymectomy: a retrospective cohort study
Source: Front Surg. 2026 Jun 22;13:1802382. doi: 10.3389/fsurg.2026.1802382 (PMC13333515; doi:10.3389/fsurg.2026.1802382)
Supplement: Supplementary file 1 [file Table1.docx]

**Supplementary Table S1.** Clinicopathological and surgical characteristics of patients with thymoma recurrence

| Patient | Surgical approach | Age (years) | Sex | Myasthenia gravis | Maximum tumor diameter (cm) | Histological type | Masaoka-Koga stage | Time to recurrence (months) | Recurrence detection method | Site of recurrence | Management |
| --- | --- | --- | --- | --- | --- | --- | --- | --- | --- | --- | --- |
| 1 | Subxiphoid approach | 56 | Female | Yes | 3.8 | B2 thymoma | II | 14 | Chest CT | Pleural lesion | Surgical resection |
| 2 | Subxiphoid approach | 49 | Male | No | 4.2 | B3 thymoma | II | 18 | Chest CT + PET/CT | Anterior mediastinum | Radiotherapy |
| 3 | Lateral intercostal approach | 61 | Male | Yes | 3.5 | B2 thymoma | II | 16 | Chest CT | Pleural lesion | Surgical resection |
| 4 | Lateral intercostal approach | 53 | Female | No | 4.0 | B1 thymoma | II | 20 | Chest CT + biopsy | Mediastinal soft tissue | Radiotherapy |
